# Total costs of basal or premixed insulin treatment in 5077 insulin-naïve type 2 diabetes patients: register-based observational study in clinical practice

**DOI:** 10.1186/s40842-015-0017-1

**Published:** 2015-12-15

**Authors:** Ann-Marie Svensson, Vincent Lak, MirNabi Pirouzi Fard, Björn Eliasson

**Affiliations:** 1 Center of Registers in Region Västra Götaland, Göteborg, Sweden; 2 Department of Medicine, Sahlgrenska University Hospital, University of Gothenburg, S-413 45 Göteborg, Sweden

**Keywords:** Type 2 diabetes, Basal insulin, Cost, Insulin detemir, Insulin glargine, Neutral protamine hagedorn, Premixed insulin

## Abstract

**Background:**

To investigate the costs of treatment with basal insulin (insulin NPH [NPH], insulin glargine [IG], insulin determir [IG]), and premixed insulin (PM) in routine clinical care.

**Methods:**

Cohort study based on data from the Swedish National Diabetes Register, including 5077 insulin-naïve men and women with type 2 diabetes, resident in a distinct geographical region of Sweden. Patients were included between 1 July 2006 and 31 December 2009 and followed for 12 months. All drug- and healthcare-related costs, stratified by diabetes-related or non-diabetes care contacts, were quantified and compared to baseline.

**Results:**

Initiation of insulin treatment generally entails increased diabetes-related health care contacts and treatment costs, and decrease in health care costs. The median changes in costs were generally smaller than the mean changes, reflecting great variations between patients. The treatment costs were higher for IG, ID and PM compared with NPH, although higher age, history cardiovascular disease and diabetes complications as well as higher diabetes-related and other treatment costs were independent predictors. Overall, only PM (but not IG or ID) were associated with higher diabetes-related health care costs, although these were also independently predicted by cardiovascular morbidity and markers of complicated diabetes.

**Conclusions:**

This study demonstrates that the initiation of insulin in patients with type 2 diabetes in clinical practice leads to increased health care contacts, overall and treatment costs, but also generally results in a decrease in health care costs. The diabetes-related treatment cost was lowest using NPH insulin but only premixed insulin was associated with higher diabetes-related health care costs than NPH.

**Electronic supplementary material:**

The online version of this article (doi:10.1186/s40842-015-0017-1) contains supplementary material, which is available to authorized users.

## Background

Insulin treatment is commonly used in patients with type 2 diabetes when lifestyle changes and oral hypoglycemic agents (OHA) fail to achieve adequate glycemic control [1, 2]. The medium long-acting NPH (neutral protamine Hagedorn) given at bedtime has been a common first-hand choice, but a long-acting insulin analogue (insulin glargine (IG) or insulin detemir (ID)) is frequently used, particularly in patients experiencing nocturnal hypoglycaemia. Pre-mixed insulin (PM), usually administered twice daily, is another useful treatment option [3].

The clinical effects of these treatment alternatives have been evaluated in randomized clinical trials (RCT) and meta-analyses, and recently also in clinical practice [4, 5]. Overall there are no major differences in the effects on glycemic control, but there can be differences in weight effects, hypoglycemia and insulin doses as well as in persistence. We recently studied the clinical effects in 5077 insulin-naïve type 2 diabetes patients in a geographically distinct region of Sweden (Region Västra Götaland), who initiated treatment on NPH, IG, ID or PM [6]. The different insulin regimens were found to be equally effective in lowering HbA1c, but PM required 59 % higher and ID 25 % higher insulin doses to achieve a similar HbA1c reduction as NPH. PM was also associated with a significantly greater increase in BMI compared with NPH, and a small but higher number of patients experiencing severe hypoglycemia than the other treatment groups.

The high costs associated with diabetes care have long been recognized, but the number of published studies in this area is low. A recent report based on managed care administrative data in the U.S.A. described in detail the total health care costs for patients with type 2 diabetes [7]. The strongest predictors of high costs for the patients were obesity, comorbidities and hospitalization, but also progression to insulin therapy. There are also very few studies addressing the total costs associated with different insulin regimens, although development programs (RCTs) for new pharmaceutical agents also often include health economic analyses [8]. In clinical practice, the addition of IG, compared with PM, to treatment with oral hypoglycemic agents (OHA) was recently examined, showing better persistence and lower costs [9].

There are no studies available comparing costs of different insulin regimens when added to OHA in unselected cohorts in clinical practice. The aim of this study was therefore to examine health care utilization and costs in our recent study with 5,077 insulin-naïve type 2 diabetes patients, after starting treatment with NPH, IG, ID or PM [6].

## Methods

The overall design of this study has recently been described in detail [6]. To summarize, we linked data from four national health registers: the Swedish National Diabetes Register (NDR; clinical data), the Prescribed Drug Register (pharmacological agents, doses), the Cause of Death Register, and the Regional Claims Database (VEGA). The latter contains diagnoses (International Classification of Diseases [ICD]-10 and Diagnosis Related Groups [DRG] codes), procedures performed (Nordic Medico-Statistical Committee [NOMESCO] and local procedure codes) and hospital lengths of stay for inpatient, outpatient, primary, and private care for all inhabitants in the Region Västra Götaland.

### Ethics, consent and permissions

All included patients have agreed by informed consent to be registered before inclusion. The Ethics Review Board at the University of Gothenburg approved the study.

### Patients, study period, follow-up and censoring

We included insulin-naïve patients with type 2 diabetes, at least 18 years of age. The study period was between 1 July 2005 and 31 December 2010. Patients were not allowed to fill a prescription of insulin from 1 July 2005 to 30 June 2006 to ensure they were previously untreated (data from the Prescribed Drug Register). Patients were required to have their first prescription of insulin filled (index date) between 1 July 2006 and 31 December 2009 to allow for 1 year of follow-up. Patients were thus followed for 12 months or until the occurrence of a censoring event. The mean number days of follow-up were similar in the four treatment groups (352 ± 47 NPH, 358 ± 37 IG, 351 ± 51 ID and 352 ± 47 PM) [6]. Start of follow-up was defined as the date of the first filled insulin prescription (index date) in each patient. In order to ensure continuous insulin use, at least three filled prescriptions of the initiated insulin were required during the follow-up period. Censoring events included a filled prescription of a new type of insulin, death or move out of the Region Västra Götaland.

### Costs

*Treatment costs* (drug-related costs) were retrieved from the Prescribed Drug Register, which has full, nation-wide coverage of all transactions that are made at pharmacies in Sweden, including all drugs (ATC codes A-V) and technical aids (ATC codes W-Y). We used data between 2005-07-01 and 2010-12-31.

*Healthcare costs* were estimated by using the associated DRG-codes (data from VEGA). To make data comparable, Swedish national DRG-weight lists and costs for 2010 were used (National Board of Health and Welfare, http://www.socialstyrelsen.se/english). VEGA also provided data on outlier costs, which were considered when estimating the final cost per care contact. For outdated DRG-codes, old DRG-weight lists provided by the Swedish National Board of Health and Welfare were used to extract weights, which were used in combination with the 2010 DRG cost. Further, psychiatric DRG-codes were flagged, and the clinic setting where the care contacts occurred, were used to determine whether DRG-weights from the inpatient DRG-list or the psychiatric DRG-list should be applied. For primary care, costs were determined based on the caregiver, where physicians were assumed to have a three times higher rate than other caregivers (e.g. nurses, physiotherapists, etc.). This corresponded to a 1840 SEK cost for a physicians visit, and a 615 SEK cost for all other primary care visits. The approximate value of 100 SEK is currently 12 US dollars (August 2015).

We studied all drug- and health care-related costs, stratified by diabetes-related or non-diabetes care contacts. All costs 12 months before initiation and during follow-up of insulin treatment were recorded. A correlation analysis with costs before and after insulin treatment initiation was performed, to determine whether incremental analysis or pre-index cost adjustment should be undertaken. If the correlation exceeded 0.5, an incremental analysis whereby the prior (baseline) costs are subtracted from the costs observed during follow-up, was recommended. Otherwise, the pre-index costs were included as a covariate in the regression model. Costs are presented in 2010 SEK value after adjusting for consumer price indices provided by Statistics Sweden (www.scb.se/en).

Costs were stratified by whether they were diabetes-related or not, in a mutually exclusive manner, including anti-diabetic treatment costs as well as costs of cardiovascular risk factor treatments (antihypertensive and lipid-lowering treatment, platelet aggregation inhibitors). We also identified all diabetes-related care contacts and their associated costs through the ICD-10 codes E11*, E13*, and E14* (regardless of diagnosis position), as well as cardiovascular disease costs, such as myocardial infarction/ischaemic heart disease (I20-I25), atrial fibrillation (I48), congestive heart failure (I50*), and stroke (I61, I63, I64, I67.9).

### Statistical methods

Baseline characteristics are presented as means ± 1 standard deviation (SD) or medians for continuous variables and frequencies for categorical variables with crude significance levels for differences between the groups, when analysed using ANOVA or *χ* ^2^ test. For continuous variables with non-normal distribution a Kruskal-Wallis test was performed. All continuous outcome variables were explored for their distribution.

We used generalized linear modeling (GLM) after log transformation to assess potential predictors of diabetes-related health care contacts, health care costs and treatment costs. For each outcome, three models were explored and presented; firstly, unadjusted where outcome was as a function of insulin groups, secondly, including covariates with few missing values (age, gender, level of income, diabetes duration, history of CVD, history of diabetes complications, previous OHA use, and follow-up time), and finally a fully adjusted model including age, gender, level of income, diabetes duration, history of CVD, history of diabetes complications, previous OHA use, follow-up time, pre-index HbA1c, pre-index BMI and weight. Pre-index diabetes-related costs were included as a covariate to account for the patient’s type 2 diabetes history and severity. Further, post-index other costs (including cardiovascular costs) were included in the model as a covariate to account for the overall disease burden (comorbidities) of the population, which in turn might affect post-index diabetes-related costs.

Statistical analyses were performed in R (R Foundation for Statistical Computing) or SAS V.9.3 (SAS Institute, Cary, North Carolina, USA). A two-sided *p* value <0.05 was considered statistically significant.

## Results

As previously reported, 5077 insulin-naïve patients with type 2 diabetes were included in the study [6]. The patients were mostly initiated on NPH (49 %) or PM (34 %), while 13 % and 3 % were initiated on IG and ID. To summarize, there were modest but significant reductions in glycemic control (HbA1c) for patients treated with NPH, IG, ID or PM during one year of follow-up, but the effects of the different regimens did not differ. The weight-adjusted daily insulin doses with ID and PM were 59 % and 25 % higher to achieve similar HbA1c when compared with patients treated with NPH, while the patients treated with PM gained more weight compared with patients treated with other insulin regimens. The recorded number of patients experiencing a hypoglycaemic event was low (only 26 patients in total), but occurred predominantly in patients treated with PM. The mean number of days of follow-up was highest in patients initiating IG.

The numbers of visits in health care before and after starting the insulin treatment are given in Table 1. There was an increase in the number of visits for all four analyzed insulin regimens during the follow-up period. For diabetes-related visits, small differences in the mean number of visits during the pre-index period were observed, such that IG and PM patients had slightly more visits. During the follow-up period, NPH patients had the highest mean number of diabetes-related health care contacts, while ID had the lowest. When diabetes-related care contacts including cardiovascular comorbidities were evaluated, PM had the highest mean number of contacts during the pre-index period, while during the follow-up period, IG, PM, and NPH patients all had close to three contacts. For other care contacts, PM and IG patients had the highest frequency during both the pre-index and the post-index period. For the patients who did have a change in number of contacts (N = 4239), there was no significant difference between the different treatment groups (Additional file 1: Table S1). Adjusting for several covariates emphasized this result, although lower age and use of OHA were independent predictors of less health care contacts. A history of cardiovascular disease, diabetes complications, higher pre-index diabetes-related and other health care costs were independent predictors of more health care contacts.

**Table 1:** Number of contacts with the health-care before and after starting insulin treatment

	Diabetes-related contacts		Diabetes-related contacts including cardiovascular		Other contacts	
	Pre-index	Post-index	Pre-index	Post-index	Pre-index	Post-index
Treatment	Mean ± SD	Mean ± SD	Mean ± SD	Mean ± SD	Mean ± SD	Mean ± SD
NPH	2.0 ± 1.8	2.7 ± 2.8	2.2 ± 2.4	3.1 ± 3.7	11.8 ± 15.4	13.9 ± 16.1
IG	2.1 ± 2.4	2.6 ± 3.1	2.3 ± 2.7	2.9 ± 3.3	12.6 ± 14.7	15.0 ± 16.2
ID	1.9 ± 2.1	2.4 ± 2.4	2.1 ± 2.4	2.6 ± 2.6	12.0 ± 13.8	14.2 ± 16.0
PM	2.1 ± 1.9	2.6 ± 2.5	2.4 ± 2.3	3.0 ± 3.3	13.1 ± 15.5	14.8 ± 15.5
*p*-values	0.003	0.044	0.00004	0.022	0.00002	0.0009

*NPH* Neutral protamine Hagedorn, *IG* insulin glargine, *ID* insulin determir, *PM* premixed insulin, *SD* standard deviation

The costs of health care and treatments before and after starting the insulin treatment are given in Table 2. Diabetes-related costs varied substantially between the treatment groups for all pre-index and post-index treatment and health care costs. The mean pre-index health care costs were considerably higher in the PM patients at (SEK 30 990), compared with SEK 22 861 and SEK 19 674 for IG and NPH groups, and SEK 9544 for ID, reflecting differences in clinical characteristics. The mean pre-index treatment costs were around 50 % higher for IG and ID compared with PM and NPH. The health care costs during follow-up were lower for all groups but the ID group, with a similar magnitude of decrease for IG and NPH, while the PM showed the largest decrease. Post-index diabetes-related treatment costs were higher for all groups, compared to pre-index treatment costs, in line with the expectations of a more intense treatment regime after insulin initiation. As expected, the IG and ID still had higher treatment costs, while the proportional cost increase compared to PM and NPH was smaller for IG, at 30 %, compared to 50 % for ID.

**Table 2:** Costs (SEK) of health care and treatments before and after starting the insulin treatment

	Pre-index		Post-index		Increment		Net cost effect
	Health care	Treatment	Health care	Treatment	Health care	Treatment	
Treatment	Mean/Median	Mean/Median	Mean/Median	Mean/Median	Mean/Median	Mean/Median	Mean/Median
NPH	19,674/3,228	2,455/1,587	16,570/3,240	8,191/6,840	−3,104/0	5,736/4,528	2,632/4,528
IG	22,861/3,457	2,857/1,959	19,746/3,143	10,818/9,343	−3,115/0	7,962/6,551	4,847/6,551
ID	9,544/2,458	3,041/1,608	11,402/2,647	12,351/10,706	1,858/0	9,310/7,690	11,168/7,690
PM	30,990/4,806	2,155/1,430	22,441/3,681	8,617/7,877	−8,549/-265	6,462/5,972	−2,087/5,707
*p*-values	<0.0001	<0.0001	0.0001	<0.0001	0.0011	<0.0001	

*NPH* Neutral protamine Hagedorn, *IG* insulin glargine, *ID* insulin determir, *PM* premixed insulin, *SD* standard deviation. The approximate value of 100 SEK is currently 12 US dollars (August 2015)

For diabetes-related costs where also cardiovascular costs were included (Table 3), similar patterns as for diabetes-related costs were observed. The main differences were that pre-index treatment costs were more similar between groups when CVD treatment was taken into account, and that all treatment groups showed cost decreases when comparing pre- and post-index health care costs.

**Table 3:** Costs (SEK) of health care and treatments (including cardiovascular costs) before and after starting the insulin treatment

	Pre-index		Post-index		Increment		Net cost effect
	Health care	Treatment	Health care	Treatment	Health care	Treatment	
Treatment	Mean/Median	Mean/Median	Mean/Median	Mean/Median	Mean/Median	Mean/Median	Mean/Median
NPH	24,791/3,681	3,449/2,398	19,390/3,681	9,279/7,879	−5,401/0	5,831/4,659	430/4,659
IG	25,197/3,681	3,920/2,679	23,539/3,681	11,967/10,670	−1,658/0	8,047/6,569	6,389/6,569
ID	17,981/2,613	3,815/2,194	13,070/2,955	13,143/11,306	−4,912/0	9,328/7,808	4,416/7,808
PM	36,724/6,366	3,183/2,259	26,388/4,262	9,744/8,800	−10,336/-773	6,560/6,010	−3,776/5,237
*p*-values	<0.0001	<0.0012	<0.0001	<0.0001	0.0022	<0.0001	

*NPH* Neutral protamine Hagedorn, *IG* insulin glargine, *ID* insulin determir, *PM* premixed insulin, *SD* standard deviation. The approximate value of 100 SEK is currently 12 US dollars (August 2015)

The median costs for both health care and treatment in both Tables 2 and 3 were consistently lower than the mean costs, reflecting an asymmetrical cost distribution among the patients with a few patients with very high costs. The discrepancies between the median and the mean costs were more pronounced in the health care costs than the treatment costs. The median increment health care cost was 0 for NPH, IG and ID. For PM there was a small decrement in median health care costs.

For other pre-index health care costs (Table 4), the PM patients consistently showed highest mean costs and ID patients lower costs, and this pattern was repeated also for pre-index treatment costs. For post-index costs, the IG and PM groups showed the highest mean costs, which were also seen for post-index treatment costs (*p* < 0.01). Overall, the differences in incremental other costs between insulin groups were non-significant, while the patterns indicated a moderate increase for treatment costs for all groups.

**Table 4:** Other costs before and after starting the insulin treatment

	Pre-index		Post-index		Increment		Net cost effect
	Health care	Treatment	Health care	Treatment	Health care	Treatment	
Treatment	Mean/Median	Mean/Median	Mean/Median	Mean/Median	Mean/Median	Mean/Median	Mean/Median
NPH	25,417/9,215	5,209/2,445	24,173/10,439	6,287/2,926	−1,244/755	1,078/125	−166/880
IG	23,533/10,341	5,576/2,784	28,096/12,447	7,063/3,267	4,563/1,225	1,486/224	6,049/1,449
ID	20,297/9,205	4,515/1,455	22,313/10,652	5,300/1,819	2,016/1,717	785/160	2,801/1,877
PM	28,423/10,752	6,380/3,150	26,880/12,191	7,549/3,877	−1,543/883	1,169/283	−374/1,166
*p*-values	0.0054	<0.00001	0.0010	<0.00001	0.55	0.052	

*NPH* Neutral protamine Hagedorn, *IG* insulin glargine, *ID* insulin determir, *PM* premixed insulin, *SD* standard deviation. The approximate value of 100 SEK is currently 12 US dollars (August 2015)

For patients who did have a change in diabetes-related health care costs (N = 4232), IG and PM increased costs compared with NPH, whereas ID lowered costs in an unadjusted model (Additional file 1: Table S2). In the fully adjusted model only PM was associated with increased health care costs compared with NPH, but higher age, a history of cardiovascular disease, diabetes complications, and pre-index diabetes-related and other health care costs independently predicted higher costs, while previous use of OHA predicted lower diabetes-related health care costs. There were higher treatment costs with IG, ID and PM compared with NPH (Additional file 1: Table S3). ID increased treatment costs more than IG and PM in relation to NPH in both the unadjusted and the adjusted models. In the latter model, higher age, a history of cardiovascular disease, diabetes complications, use of OHA, were independent predictors of lower, while higher municipality income as well as pre-index diabetes-related and other health care costs predicted higher diabetes-related treatment costs.

## Discussion and conclusions

This observational study provides information on costs of the use different types of insulin regimens in patients with type 2 diabetes failing oral glucose-lowering treatment. The results show that although the initiation of insulin treatment generally entails increased diabetes-related health care contacts and treatment costs, it also generally results in a decrease in health care costs, especially when including costs for the treatment and care of cardiovascular diseases. The median changes in costs were generally smaller than the mean changes, reflecting great variations between patients. The treatment costs were higher for IG, ID and PM compared with NPH, although history cardiovascular disease and diabetes complications as well as higher diabetes-related and other treatment costs were independent predictors. Overall, with respect to diabetes-related health care, only PM (but not IG or ID) were associated with higher costs, although these were also independently predicted by cardiovascular morbidity and markers of complicated diabetes.

There are generally differences in clinical characteristics between the patients offered the various treatment options when additional treatment is required on top of OHA in clinical practice. Still, this project including previously presented clinical results [10], provides arguments against the use of PM due to weight gain, higher insulin doses, rates of hypoglycaemia and diabetes–related health care costs compared with the reference, treatment with NPH. The treatment costs of IG and ID are higher than NPH, but seem to offer other advantages, possibly such as ease of use and low rates of hypoglycaemia, leading to similar overall costs.

Other studies of the costs of initiating basal insulin have reached similar conclusions. A retrospective study on a U.S. population between 2001 and 2006 found that IG compared with NPH had higher drug-related costs, but no significant difference in health-care costs, although both were associated with major cost reductions [11]. Similarly, a UK retrospective primary care register study with data from 1988–2010 found that insulin analogues were associated with higher costs than NPH for the first year but after three years [12]. Contrarily, a Swiss simulation study found IG would be more cost-effective than NPH in the long term [13]. A recent systematic review and meta-analysis concluded that long acting insulin analogues are probably superior to NPH, although the difference is small for HbA1c [14]. The effects of treatment with premixed insulin have also been evaluated [9, 15], leading to careful recommendations of its use [1, 2, 16].

The present study has several strengths. The observational design allows for comparisons of the effectiveness and costs of different types of insulin, and the results are likely to be representative of clinical practice in countries with similar populations, following similar treatment guidelines [17]. All patients fulfilling the inclusion criteria have been included in the calculations, which are based on detailed information from administrative systems with complete coverage. Apart from the general limitations characteristic of observational studies, one weakness in the present study was the limited number of patients on IG and ID, particularly the latter. The lack of reliable data on non-severe hypoglycaemia (i.e., not requiring hospitalization), or patient-reported measures, are important limitations. Furthermore, Analyses did not provide a societal perspective, since neither sick-leave information (productivity gains and losses), nor direct non-medical costs, were considered [18].

This study demonstrates that the initiation of insulin in patients with type 2 diabetes in clinical practice leads to increased health care contacts, overall and treatment costs, but also generally results in a decrease in health care costs. The diabetes-related treatment cost was lowest using NPH insulin but only premixed insulin was associated with higher diabetes-related health care costs than NPH.

## Additional file

Additional file 1: Table S1. Potential predictors of diabetes-related health care contacts. **Table S2.** Potential predictors of diabetes-related health care costs. **Table S3.** Potential predictors of diabetes-related treatment costs. (DOCX 29 kb)
