# Supplementary material for: Total costs of basal or premixed insulin treatment in 5077 insulin-naïve type 2 diabetes patients: register-based observational study in clinical practice
Source: Clin Diabetes Endocrinol. 2015 Dec 15;1:17. doi: 10.1186/s40842-015-0017-1 (PMC5471692; doi:10.1186/s40842-015-0017-1)
Supplement: Additional file 1: Table S1. — Potential predictors of diabetes-related health care contacts. Table S2. Potential predictors of diabetes-related health care costs. Table S3. Potential predictors of diabetes-related treatment costs. (DOCX 29 kb) [file 40842_2015_17_MOESM1_ESM.docx]

Additional file 1

Table S1. Potential predictors of diabetes-related health care contacts

|  | N = 4,239‡ | | N = 3,881‡ | | N = 1,062‡ | | N = 3,881‡ | |  |
| --- | --- | --- | --- | --- | --- | --- | --- | --- | --- |
|  | Change* | *P*-value | Change* | *P*-value | Change* | *P*-value | Change* | *P*-value |  |
| Lantus vs. NPH | 1.214 | 0.447 | 0.998 | 0.950 | 0.981 | 0.772 | 0.982 | 0.627 |  |
| Levemir vs. NPH | 0.927 | 0.273 | 0.918 | 0.239 | 0.902 | 0.459 | 0.919 | 0.239 |  |
| Mix vs. NPH | 0.990 | 0.676 | 0.981 | 0.473 | 0.941 | 0.213 | 0.972 | 0.275 |  |
| Gender (reference: male) |  |  | 0.963 | 0.124 | 0.952 | 0.406 | 0.955 | 0.058 |  |
| Age (1 year increase) |  |  | 0.996 | <0.001 | 0.998 | 0.265 | 0.996 | <0.001 |  |
| Municipality's income (quartile) |  |  |  |  |  |  |  |  |  |
| *Q2 vs. Q1* |  |  | 0.991 | 0.831 | 0.879 | 0.085 | 0.996 | 0.918 |  |
| *Q3 vs. Q1* |  |  | 1.067 | 0.092 | 1.026 | 0.706 | 1.055 | 0.160 |  |
| *Q4 vs. Q1* |  |  | 1.019 | 0.671 | 0.935 | 0.379 | 1.011 | 0.808 |  |
| Follow-up (1 day increase) |  |  | 0.998 | <0.001 | 0.997 | <0.001 | 0.998 | <0.001 |  |
| Diabetes duration (1 year increase) | |  | 0.999 | 0.964 | 0.999 | 0.906 | 1.000 | 0.974 |  |
| History of cardiovascular disease (reference: no) | | | 1.097 | 0.001 | 1.080 | 0.106 | 1.100 | <0.001 |  |
| History of diabetes complications (reference: no) | | | 1.113 | <0.001 | 1.138 | 0.006 | 1.110 | <0.001 |  |
| Previous oral antidiabetics use (reference: no) | |  | 0.794 | <0.001 | 1.018 | 0.866 | 0.804 | <0.001 |  |
| Pre-index Diabetes-related health care costs | |  | 0.999 | <0.001 | 1.000 | <0.001 | 1.000 | <0.001 |  |
| (1 unit increase) | |  |  |  |  |  |  |  |  |
| Post-index Other health care costs (1 unit increase) | | |  |  |  |  | 1.000 | <0.001 |  |
| Pre-index HbA1c (1 unit increase) |  |  |  |  | 1.002 | 0.126 |  |  |  |
| Pre-index BMI (1 unit increase) |  |  |  |  | 1.008 | 0.344 |  |  |  |
| Pre-index Weight (1 unit increase) |  |  |  |  | 0.999 | 0.7923 |  |  |  |

* The exponentiated parameter estimate is the multiplicative effect of this level vs. the reference level (e.g., 1.04 = 4% more care contacts).

‡ Individuals with no care contacts were excluded, 4,239 corresponds to the total population with at least one diabetes-related care contact during follow-up.

Table S2. Potential predictors of diabetes-related health care costs

|  | N = 4,234‡ | | N = 3,876‡ | | N = 1,061^‡^ | | N = 3,876‡ | |  |
| --- | --- | --- | --- | --- | --- | --- | --- | --- | --- |
|  | Change* | *P*-value | Change* | *P*-value | Change* | *P*-value | Change* | *P*-value |  |
| Lantus vs. NPH | 1.321 | <0.001 | 1.195 | 0.009 | 1.253 | 0.071 | 1.061 | 0.363 |  |
| Levemir vs. NPH | 0.711 | 0.008 | 0.847 | 0.204 | 0.801 | 0.375 | 0.957 | 0.727 |  |
| Mix vs. NPH | 1.427 | <0.001 | 1.153 | 0.003 | 1.026 | 0.774 | 1.105 | 0.035 |  |
| Gender (reference: male) |  |  | 1.058 | 0.210 | 1.070 | 0.517 | 1.042 | 0.341 |  |
| Age (1 year increase) |  |  | 1.014 | <0.001 | 1.015 | <0.001 | 1.018 | <0.001 |  |
| Municipality's income (quartile) |  |  |  |  |  |  |  |  |  |
| *Q2 vs. Q1* |  |  | 0.978 | 0.769 | 0.967 | 0.810 | 1.016 | 0.834 |  |
| *Q3 vs. Q1* |  |  | 1.116 | 0.120 | 1.068 | 0.606 | 1.045 | 0.519 |  |
| *Q4 vs. Q1* |  |  | 1.174 | 0.045 | 1.047 | 0.744 | 1.047 | 0.550 |  |
| Follow-up (1 day increase) |  |  | 1.000 | 0.935 | 0.979 | 0.006 | 0.994 | <0.001 |  |
| Diabetes duration (1 year increase) | |  | 0.994 | <0.001 | 0.990 | <0.001 | 1.004 | 0.252 |  |
| History of cardiovascular disease (reference: no) | | | 1.488 | <0.001 | 1.718 | <0.001 | 1.534 | <0.001 |  |
| History of diabetes complications (reference: no) | | | 1.151 | 0.004 | 1.477 | <0.001 | 1.153 | 0.003 |  |
| Previous oral antidiabetics use (reference: no) | |  | 0.728 | <0.001 | 1.643 | 0.012 | 0.743 | <0.001 |  |
| Pre-index Diabetes-related health care costs | |  | 1.000 | <0.001 | 1.000 | <0.001 | 1.000 | <0.001 |  |
| (1 unit increase) | |  |  |  |  |  |  |  |  |
| Post-index Other health care costs (1 unit increase) | | |  |  |  |  | 1.000 | <0.001 |  |
| Pre-index HbA1c (1 unit increase) |  |  |  |  | 0.998 | 0.523 |  |  |  |
| Pre-index BMI (1 unit increase) |  |  |  |  | 1.050 | <0.001 |  |  |  |
| Pre-index Weight (1 unit increase) |  |  |  |  | 0.988 | 0.008 |  |  |  |

* The exponentiated parameter estimate is the multiplicative effect of this level vs. the reference level (e.g., 1.04 = 4% higher cost, 0.98 = 2% lower cost).

‡ Individuals with zero costs were excluded, 4,234 corresponds to the total population with a diabetes-related cost during follow-up.

Table S3. Potential predictors of diabetes-related treatment costs

|  | N = 4,234‡ | | N=3,881‡ | | N = 1,062‡ | | N = 3,881‡ | |  |
| --- | --- | --- | --- | --- | --- | --- | --- | --- | --- |
|  | Change* | *P*-value | Change* | *P*-value | Change* | *P*-value | Change* | *P*-value |  |
| Lantus vs. NPH | 1.311 | <0.001 | 1.276 | <0.001 | 1.354 | <0.001 | 1.271 | <0.001 |  |
| Levemir vs. NPH | 1.499 | <0.001 | 1.427 | <0.001 | 1.600 | <0.001 | 1.426 | <0.001 |  |
| Mix vs. NPH | 1.071 | 0.001 | 1.097 | <0.001 | 1.235 | <0.001 | 1.093 | <0.001 |  |
| Gender (reference: male) |  |  | 1.007 | 0.691 | 1.019 | 0.626 | 1.003 | 0.845 |  |
| Age (1 year increase) |  |  | 0.992 | <0.001 | 0.994 | <0.001 | 0.992 | <0.001 |  |
| Municipality's income (quartile) |  |  |  |  |  |  |  |  |  |
| *Q2 vs. Q1* |  |  | 1.084 | 0.013 | 1.017 | 0.768 | 1.085 | 0.011 |  |
| *Q3 vs. Q1* |  |  | 1.149 | <0.001 | 1.112 | 0.047 | 1.147 | <0.001 |  |
| *Q4 vs. Q1* |  |  | 1.088 | 0.010 | 1.064 | 0.278 | 1.086 | 0.017 |  |
| Follow-up (1 day increase) |  |  | 0.999 | 0.021 | 1.000 | 0.498 | 0.999 | 0.019 |  |
| Diabetes duration (1 year increase) | |  | 1.002 | 0.226 | 1.006 | 0.031 | 1.002 | 0.241 |  |
| History of cardiovascular disease (reference: no) | | | 0.966 | 0.066 | 0.969 | 0.339 | 0.964 | 0.049 |  |
| History of diabetes complications (reference: no) | | | 0.960 | 0.0264 | 0.986 | 0.645 | 0.959 | 0.022 |  |
| Previous oral antidiabetics use (reference: no) | |  | 0.747 | <0.001 | 0.785 | 0.001 | 0.753 | <0.001 |  |
| Pre-index Diabetes-related treatment costs | |  | 1.000 | <0.001 | 1.000 | <0.001 | 1.000 | <0.001 |  |
| (1 unit increase) | |  |  |  |  |  |  |  |  |
| Post-index Other treatment costs (1 unit increase) | | |  |  |  |  | 1.000 | <0.001 |  |
| Pre-index HbA1c (1 unit increase) |  |  |  |  | 1.002 | 0.099 |  |  |  |
| Pre-index BMI (1 unit increase) |  |  |  |  | 0.996 | 0.424 |  |  |  |
| Pre-index Weight (1 unit increase) |  |  |  |  | 1.005 | 0.002 |  |  |  |

* The exponentiated parameter estimate is the multiplicative effect of this level vs. the reference level (e.g., 1.04 = 4% higher cost, 0.98 = 2% lower cost).

‡ Individuals with zero costs were excluded, 4,234 corresponds to the total population with a diabetes-related cost during follow-up.
